# *Rickettsia rickettsii* Co-feeding Transmission among *Amblyomma aureolatum* Ticks

**DOI:** 10.3201/eid2411.180451

**Published:** 2018-11

**Authors:** Jonas Moraes-Filho, Francisco B. Costa, Monize Gerardi, Herbert S. Soares, Marcelo B. Labruna

**Affiliations:** Universidade Santo Amaro, São Paulo, Brazil (J. Moraes-Filho);; University of São Paulo, São Paulo (J. Moraes-Filho, F.B. Costa, M. Gerardi, H.S. Soares, M.B. Labruna);; Universidade Estadual do Maranhão, São Luís, Brazil (F.B. Costa).

**Keywords:** Rickettsia rickettsii, Amblyomma aureolatum, Rocky Mountain spotted fever, co-feeding, Brazil, vector-borne infections, guinea pigs, ticks, bacteria

## Abstract

*Amblyomma aureolatum* ticks are vectors of *Rickettsia rickettsii*, the etiologic agent of Rocky Mountain spotted fever in Brazil. Maintenance of *R. rickettsii* in nature depends on horizontal transmission along tick generations. Although such transmission is known to occur when uninfected and infected ticks feed simultaneously on susceptible animals (co-feeding systemic transmission), we investigated co-feeding nonsystemic transmission, which was based on *R. rickettsii*–infected and –uninfected *A. aureolatum* ticks feeding simultaneously on guinea pigs immune to *R. rickettsii*. Our acquisition and transmission infestations demonstrated that horizontal transmission of *R. rickettsii* by co-feeding ticks on immune hosts with no systemic infection did not occur when uninfected larvae fed distantly from infected nymphs but did occur in a few cases when uninfected larvae fed side-by-side with infected nymphs, suggesting that they shared the same feeding site. The co-feeding nonsystemic transmission type might have no epidemiologic importance for Rocky Mountain spotted fever.

The bacterium *Rickettsia rickettsii* is the etiologic agent of Rocky Mountain spotted fever or Brazilian spotted fever (*1*). In Brazil, where Brazilian spotted fever fatality rates are >50% (*2*), *R. rickettsii* is transmitted to humans by 2 tick species, *Amblyomma aureolatum* and *A. sculptum* (*3*). Although *R. rickettsii* is transovarially transmitted in ticks, the vertical transmission is not sufficient to guarantee maintenance of this bacterium in the tick population because of low rates of transovarial transmission (*A. sculptum*) (*4*) or because of a higher mortality rate for infected ticks (*A. aureolatum*) (*5*). In either case, the creation of new cohorts of infected ticks by horizontal transmission along tick generations is required for the successful establishment of *R. rickettsii* infection in the tick population (*5*).

Since the classical works of Ricketts (*6*) and subsequent rickettsiologists (*7**–**9*), horizontal transmission of *R. rickettsii* has been believed to depend chiefly on the simultaneous feeding of uninfected and infected immature ticks on susceptible animals, also called amplifying hosts (*3**,**10*). Once infested by an *R. rickettsii–*infected tick, the host develops a systemic infection (rickettsemia) lasting ≈1–3 weeks, during which time uninfected ticks acquire rickettsial infection upon feeding (*7**–**9*). After this period, the host develops an immune response that precludes new rickettsemia, even when infested again by *R. rickettsii–*infected ticks (*7**–**9*). Based on these premises, it has been proposed that each individual amplifying host will generate only 1 rickettsemia of 1–3 weeks in its lifespan; thereafter, the host becomes immune to *R. rickettsii* infection (*3**,**8**,**11*).

Niebylski et al. (*10*) reported that transmission between co-feeding ticks and by transovarial transmission might further enhance rickettsial infection rates in ticks. In this case, co-feeding refers to the simultaneous feeding of uninfected and infected immature ticks on susceptible host animals (amplifying hosts developing rickettsemia), as reported previously (*6*–*9*). In the late 1980 and the 1990s, the term co-feeding was introduced for nonsystemic transmission of tickborne viruses in vertebrate hosts (*12**–**14*). Since then, use of the term co-feeding has apparently generated confusion. Indeed, co-feeding means that >2 ticks are feeding simultaneously on the same individual host. Co-feeding ticks can result in 2 main types of horizontal transmission of pathogens: 1) systemic transmission, (e.g., *R. rickettsia*) (*10*); and 2) nonsystemic transmission, (e.g., tickborne encephalitis virus) (*13**,**14*). Because the co-feeding systemic transmission type is well known for *R. rickettsii* in ticks, we evaluated the occurrence of the co-feeding nonsystemic transmission type in an *A. aureolatum*–*R. rickettsii–*guinea pig model.

## Materials and Methods

### Tick Colony and *R. rickettsii* Infection

During 2012, we established a laboratory colony of *A. aureolatum* ticks in the laboratory from engorged females free of rickettsial infection collected on dogs in São Bernardo do Campo in the São Paulo metropolitan area of Brazil, as described (*15*). We divided this colony into 2 cohorts; 1 was experimentally infected by *R. rickettsii*, and the other remained uninfected. The infected cohort was created by allowing larvae to feed on rickettsemic guinea pigs that were intraperitoneally inoculated with the Taiaçu strain of *R. rickettsii*, as described elsewhere (*5**,**15*). Molecular analysis (detection of rickettsial DNA in postmolting ticks) and feeding on guinea pigs (successful transmission of rickettsia) showed that 100% of the ticks from this cohort were infected by *R. rickettsii* (*15*). For this study, we used nymphs from the infected cohort for rickettsia-donor feeding (for transmission of *R. rickettsii* to guinea pigs) and larvae from the uninfected cohort for acquisition feeding. The Ethical Committee in Animal Research of the Faculty of Veterinary Medicine of the University of São Paulo approved this study.

### Acquisition Infestation 1: Susceptible Guinea Pigs

Each of 6 tick-naive (with no previous tick infestation) adult male guinea pigs (guinea pigs 1–6), >5 mo old, weighing >500 g, seronegative for *R. rickettsii* 1 day before tick infestation, had 2 cotton sleeves (5-cm diameter feeding chamber) glued to its shaved dorsum, as described (*16*). The minimum distance between the 2 chambers was 3 cm. On day 0, one chamber received 50 *R. rickettsii*–infected nymphs (IN); on day 3, each of the 2 chambers received 200–300 uninfected larvae (UL). Therefore, in each of the 6 guinea pigs, UL fed with IN inside 1 chamber with the chance to share the same feeding site (Figure). This condition was possible because *A. aureolatum* nymphs take 5–9 days to complete engorgement on guinea pigs (*17*). In the second chamber, UL fed physically separated from IN. All infested animals had their temperature rectally measured daily from the day of infestation (day 0) to 21 days postinfestation (dpi). Guinea pigs were considered febrile if rectal temperature reached >39.5°C. To prevent deaths of these animals, they were treated with a single intramuscular dose of doxycycline (20 mg/kg) at the second febrile day. All animals were tested for seroconversion to *R. rickettsii* antigens at 21 dpi by immunofluorescence assay, as described (*18*). Animals were considered seronegative if their serum IgG was not reactive at the 1:64 dilution. If serum was reactive at the 1:64 dilution, it was titrated to determine endpoint titers to *R. rickettsii*. Each day we recovered naturally detached engorged larvae and nymphs from the feeding chambers and immediately placed them in an incubator (23°C, 95% relative humidity) for molting. From the resultant molted nymphs and adult ticks, a random sample was submitted to DNA extraction by the guanidine isothiocyanate-phenol technique (*19*) 10–20 days after molting and tested by a Taqman real-time PCR targeting the rickettsial *gltA* gene, as described (*20*), to determine the proportion of ticks that contained rickettsial DNA. The sensitivity of this PCR was determined to be 1 DNA copy of *R. rickettsii* (*20*).

**Figure:**
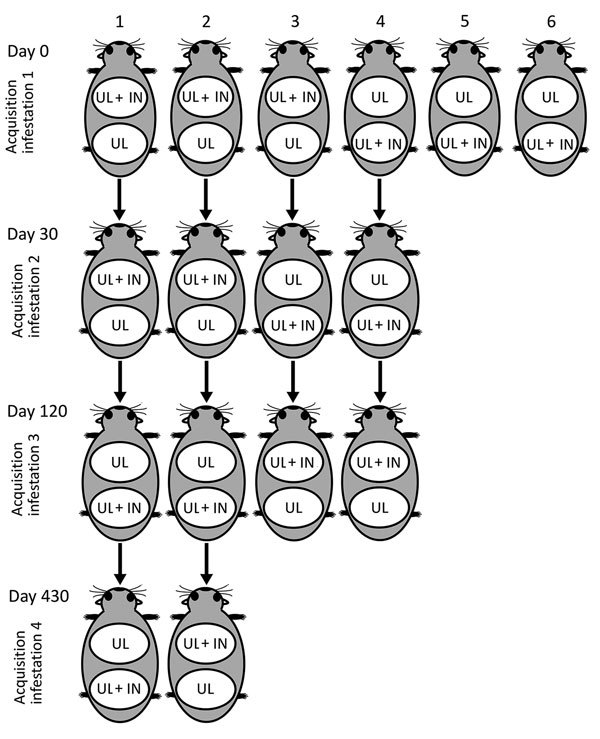
Experimental procedures to evaluate co-feeding transmission of *Rickettsia rickettsii* among *Amblyomma aureolatum* ticks on 6 guinea pigs (as numbered) subjected to up to 4 consecutive infestations at 0, 30, 120, and 430 days postinfestation, Brazil. Each guinea pig in each acquisition infestation had 2 cotton sleeves (feeding chambers) glued to its shaved back; the 2 chambers each received 200–300 UL, whereas only 1 chamber received 50 *R. rickettsii* IN. White oval indicates feeding chamber. IN, infected nymph; UL, uninfected larvae.

### Acquisition Infestations 2, 3, and 4: Immune Guinea Pigs

We used 4 guinea pigs from acquisition infestation 1 (guinea pigs 1–4) for acquisition infestations 2 and 3, conducted 30 and 120 days, respectively, after acquisition infestation 1. We used guinea pigs 1 and 2 in acquisition infestation 4, conducted 330 days after acquisition infestation 1. In all cases, each animal had 2 feeding chambers, which received *R. rickettsii* IN, UL, or both, as described for acquisition infestation 1 (Figure). We collected a blood sample on the infestation day to determine the endpoint titer to *R. rickettsii* when guinea pigs received the *R. rickettsii*–IN. Measurements of rectal temperature, recovery of engorged ticks, and molecular tests of ticks were performed as described for acquisition infestation 1.

### Transmission Infestations

Unfed nymphs derived from engorged larvae in acquisition infestations 1–4 were used to infest 29 naive adult guinea pigs (guinea pigs 11–39), >3 mo old, weighing >300 g. We considered horizontal transmission of *R. rickettsii* as successful only if these nymphs transmitted rickettsia to these guinea pigs. For this purpose, each guinea pig was prepared with a single feeding chamber that received 20 unfed nymphs derived from engorged larvae from a single feeding chamber in each of the acquisition infestations. This procedure evaluated rickettsial transmission by nymphs that had fed as larvae in the same chamber with IN and by nymphs that had fed as larvae in a chamber physically separated from IN. Procedures for rectal temperature and seroconversion were as described previously. No febrile guinea pig was treated with doxycycline in these transmission infestations; therefore, if infested guinea pigs died before the 21 dpi, a spleen fragment was submitted to DNA extraction by using the DNeasy Blood and Tissue Kit (QIAGEN, Chatsworth, CA, USA) and tested by the same PCR protocol referenced earlier. Naturally detached engorged nymphs were allowed to molt to adults and then tested by real-time PCR as described for acquisition ticks.

## Results

### Acquisition Infestation 1

All 6 guinea pigs (nos. 1–6) manifested fever, starting at 5–9 dpi. Larval infestation was done 3 days after the nymphal infestation; therefore, the larval feeding period (4–7 days) overlapped with nymphal feeding (5–9 days) and with febrile periods of the 6 guinea pigs. On the second day of fever, each guinea pig was treated with doxycycline, which resolved fever in 48 h, when most of the larvae had already completed feeding. Blood samples collected at 21 dpi showed seroconversion to *R. rickettsii* (endpoint titers 8,192–65,536). From the engorged larvae and nymphs collected from guinea pigs 1–6, unfed nymphs and adults, respectively, were tested by PCR after molting (Table 1). In all cases, 100% of the ticks contained rickettsial DNA, regardless of the feeding chamber (Table 1). This result demonstrated that *A. aureolatum* larvae acquired rickettsial DNA by feeding either separated from IN (feeding chamber UL) or by feeding together with IN within the same chamber (feeding chamber UL + IN).

**Table 1:** *Rickettsia rickettsii* acquisition infestation 1 with *Amblyomma aureolatum* ticks on 6 guinea pigs, Brazil*

Guinea pig	Fever onset, dpi (maximum temperature, °C)	IFA endpoint titer at 21 dpi†	Feeding chamber‡	PCR on ticks after molting, no. infected/no. tested (% infected)	
						Unfed nymphs	Unfed adults
1	6 (40.3)	65,536	UL + IN	10/10 (100)	10/10 (100)
			UL	10/10 (100)	
2	8 (40.0)	65,536	UL + IN	9/9 (100)	10/10 (100)
			UL	10/10 (100)	
3	8 (40.5)	8,192	UL + IN	15/15 (100)	5/5 (100)
			UL	15/15 (100)	
4	5 (40.7)	65,536	UL	15/15 (100)	
			UL + IN	15/15 (100)	8/8 (100)
5	9 (40.0)	16,384	UL	15/15 (100)	
			UL + IN	15/15 (100)	8/8 (100)
6	7 (40.4)	16,384	UL	15/15 (100)	
			UL + IN	15/15 (100)	6/6 (100)

*Each guinea pig was infested on day 0 with *R. rickettsii* IN and on day 3 with UL. Recovered engorged larvae and nymphs were allowed to molt to nymphs and adult ticks, respectively, which were tested by real-time PCR for presence of rickettsial DNA. dpi, days postinfestation; IFA, immunofluorescence assay; IN, infected nymphs; UL, uninfected larvae. †Blood was collected at 21 dpi and tested by IFA with *R. rickettsii* antigens. ‡Tick infestations were performed on 2 feeding chambers glued to the shaved back of each guinea pig, 1 chamber receiving IN and UL, the other receiving only UL (Figure).

### Acquisition Infestation 2

This infestation was performed on guinea pigs 1–4, at 30 days after acquisition infestation 1, when their endpoint titers to *R. rickettsii* were 32,768–65,536. None of the 4 guinea pigs manifested fever (Table 2). Unfed nymphs and adults that molted from engorged larvae and nymphs, respectively, were tested by PCR. All adult ticks (derived from *R. rickettsii*–IN) contained rickettsial DNA. None of the unfed nymphs derived from the feeding chamber UL (UL feeding physically separated from IN) contained rickettsia. Similarly, for 2 guinea pigs (nos. 1, 2), none of the unfed nymphs derived from engorged larvae that fed in feeding chamber UL + IN (UL feeding together with IN) contained rickettsia; in contrast, for guinea pigs 3 and 4, 17.7%–33.3% of the unfed nymphs derived from feeding chamber UL + IN contained rickettsial DNA. This result demonstrated that *A. aureolatum* larvae did not acquire rickettsial DNA by feeding separated from IN (feeding chamber UL). When feeding together with IN within the same chamber (feeding chamber UL + IN), for guinea pigs 3 and 4, a minority of *A. aureolatum* larvae acquired rickettsial DNA, whereas for guinea pigs 1 and 2, *A. aureolatum* larvae did not acquire rickettsial DNA.

**Table 2:** *Rickettsia rickettsii* acquisition infestation 2 with *Amblyomma aureolatum* ticks on 4 guinea pigs 30 days after acquisition infestation 1, Brazil*

Guinea pig	Temperature range, °C	IFA endpoint titer at day 0†	Feeding chamber‡	PCR on ticks after molting, no. infected/no. tested (% infected)	
				Unfed nymphs	Unfed adults
1	No fever to 38.8	32,768	UL + IN	0/10 (0)	2/2 (100)
			UL	0/10 (0)	
2	No fever to 38.4	32,768	UL + IN	0/10 (0)	3/3 (100)
			UL	0/10 (0)	
3	No fever to 39.1	32,768	UL	0/30 (0)	
			UL + IN	10/30 (33)	3/3 (100)
4	No fever to 39.2	65,536	UL	0/30 (0)	
			UL + IN	5/30 (17)	3/3 (100)

*Each guinea pig was infested on day 0 with *R. rickettsii* IN and on day 3 with UL. Recovered engorged larvae and nymphs were allowed to molt to nymphs and adult ticks, respectively, which were tested by real-time PCR for presence of rickettsial DNA. dpi, days postinfestation; IFA, immunofluorescence assay; IN, infected nymphs; UL, uninfected larvae. †Blood was collected at day 0 (30 days after acquisition infestation 1) and tested by IFA with *R. rickettsii* antigens. ‡Tick infestations were performed on 2 feeding chambers glued to the shaved back of each guinea pig, 1 chamber receiving IN and UL, the other receiving only UL (Figure).

### Acquisition Infestation 3

This infestation was performed on guinea pigs 1–4 at 120 and 90 days after acquisition infestations 1 and 2, respectively, when their endpoint titers to *R. rickettsii* were 4,096–16,384. None of the 4 animals manifested fever (Table 3). Unfed nymphs and adults that molted from engorged larvae and nymphs, respectively, were tested by PCR. All adult ticks (derived from *R. rickettsii*–IN) contained rickettsial DNA. In guinea pigs 1 and 2, none of the unfed nymphs derived from both feeding chambers (UL or UL + IN) contained rickettsial DNA. For guinea pigs 3 and 4, 10%–28.6% of the unfed nymphs derived from feeding chamber UL + IN contained rickettsial DNA, as did 12.0%–16.7% of the unfed nymphs derived from feeding chamber UL (Table 3). This result demonstrated that, for 2 animals, *A. aureolatum* larvae did not acquire rickettsial DNA by feeding either separated from IN (feeding chamber UL) or together with IN (feeding chamber UL + IN). In 2 other animals, a minority of *A. aureolatum* larvae acquired rickettsial DNA by feeding either separated from IN (feeding chamber UL) or together with IN (feeding chamber UL + IN).

**Table 3:** *Rickettsia rickettsii* acquisition infestation 3 with *Amblyomma aureolatum* ticks on 4 guinea pigs 120 days after acquisition infestation 1, Brazil*

Guinea pig	Temperature range, °C	IFA endpoint titer†	Feeding chamber‡	PCR on ticks after molting, no. infected/no. tested (% infected)	
						Unfed nymphs	Unfed adults
1	No fever to 38.9	16,384	UL	0/13 (0)	
			UL + IN	0/13 (0)	5/5 (100)
2	No fever to 39.2	8,192	UL	0/13 (0)	
			UL + IN	0/13 (0)	7/7 (100)
3	No fever to 38.9	4,096	UL + IN	2/7 (29)	8/8 (100)
			UL	3/25 (12)	
4	No fever to 38.5	4,096	UL + IN	3/30 (10)	4/4 (100)
			UL	5/30 (17)	

*Each guinea pig was infested on day 0 with *R. rickettsii* IN and on day 3 with UL. Recovered engorged larvae and nymphs were allowed to molt to nymphs and adult ticks, respectively, which were tested by real-time PCR for presence of rickettsial DNA. dpi, days postinfestation; IFA, immunofluorescence assay; IN, infected nymphs; UL, uninfected larvae. †Blood was collected at day 0 (120 and 90 days after acquisition infestations 1 and 2, respectively) and tested by IFA with *R. rickettsii* antigens. ‡Tick infestations were performed on 2 feeding chambers glued to the shaved back of each guinea pig; 1 chamber receiving IN and UL, the other receiving only UL (Figure).

### Acquisition Infestation 4

This infestation was performed on guinea pigs 1 and 2 at 430, 400, and 310 days after acquisition infestations 1, 2, and 3, respectively, when their endpoint titers to *R. rickettsii* were 512–4,096. Neither animal manifested fever (Table 4). Unfed nymphs and adults that molted from engorged larvae and nymphs, respectively, were tested by PCR. All adult ticks (derived from *R. rickettsii*–IN) contained rickettsial DNA. No engorged larvae were recovered from guinea pig 1; therefore, there was no molted nymph to be tested. In guinea pig 2, 8.4% of the unfed nymphs derived from feeding chamber UL + IN contained rickettsial DNA, as did 12.5% of the unfed nymphs derived from feeding chamber UL (Table 4).

**Table 4:** *Rickettsia rickettsii* acquisition infestation 4 with *Amblyomma aureolatum* ticks on 2 guinea pigs 430 days after acquisition infestation 1, Brazil*

Guinea pig	Temperature range, °C	IFA endpoint titer†	Feeding chambers‡	PCR on ticks after molting, no. infected/no. tested (%)	
				Unfed nymphs	Unfed adults
1	No fever to 38.7	4,096	UL	ND	
			UL + IN	ND	2/2 (100)
2	No fever to 38.7	512	UL	2/16 (13)	
			UL + IN	1/12 (8)	5/5 (100)

*Each guinea pig was infested on day 0 with *R. rickettsii* IN and on day 3 with UL. Recovered engorged larvae and nymphs were allowed to molt to nymphs and adult ticks, respectively, which were tested by real-time PCR for presence of rickettsial DNA. dpi, days postinfestation; IFA, immunofluorescence assay; IN, infected nymphs; ND, not done because very few engorged larvae were recovered from the animal; UL, uninfected larvae. †Blood was collected at day 0 (430, 400, and 310 days after acquisition infestations 1, 2, and 3, respectively) and tested by IFA with *R. rickettsii* antigens. ‡Tick infestations were performed on 2 feeding chambers glued to the shaved back of each guinea pig, 1 chamber receiving IN and UL, the other receiving only UL (Figure).

### Transmission Infestations

Nymphs from acquisition infestation 1, which fed as larvae on febrile guinea pigs, were used to infest 7 guinea pigs (nos. 11–17). In all animals, fever developed that started 6–7 dpi. Two animals died during the febrile period, and their spleens contained rickettsial DNA. The remaining 5 guinea pigs seroconverted for *R. rickettsii* with endpoint titers of 65,536. Engorged nymphs recovered from these guinea pigs molted to adults, all of which contained rickettsial DNA (Table 5).

**Table 5:** Transmission infestations on 29 naive guinea pigs infested with *Amblyomma aureolatum* nymphs derived from larvae that had co-fed with *Rickettsia rickettsii–*infected nymphs on 6 guinea pigs during acquisition infestations, Brazil*

Origin of nymphs				Transmission infestation					PCR on unfed adult ticks after molting from engorged nymphs, no. infected ticks/no. tested ticks (% infection)
AI	Guinea pig†	Feeding chamber†		Guinea pig	Fever onset, dpi	Maximum temperature, °C	Died	IFA endpoint titer‡	
1	3	UL		11	6	39.6	No	65,536	2/2 (100)
	4	UL		12	7	40.7	Yes§	ND	8/8 (100)
	5	UL		13	7	40.4	No	65,536	7/7 (100)
	6	UL		14	7	40.7	No	65,536	14/14 (100)
	3	UL + IN		15	6	40.9	No	65,536	6/6 (100)
	4	UL + IN		16	6	40.3	Yes§	ND	6/6 (100)
	5	UL + IN		17	7	40.6	No	65,536	5/5 (100)
2	1	UL		18	No fever–38.6		No	<64	0/10 (0)
	2	UL		19	No fever–39.1		No	<64	0/10 (0)
	3	UL		20	No fever–39.1		No	<64	0/15 (0)
	3	UL		21	No fever–38.7		No	<64	0/12 (0)
	4	UL		22	No fever–38.8		No	<64	0/8 (0)
	4	UL		23	No fever–39.4		No	<64	0/17 (0)
	1	UL + IN		24	No fever–38.8		No	<64	0/10 (0)
	2	UL + IN		25	No fever–39.4		No	<64	0/10 (0)
	3	UL + IN		26	8–40.6		No	16,384	11/12 (92)
	3	UL + IN		27	13–40.2		No	65,536	8/10 (80)
	4	UL + IN		28	No fever–38.8		No	<64	0/10 (0)
	4	UL + IN		29	No fever–39.4		No	<64	0/10 (0)
3	1	UL		30	No fever–39.3		No	<64	0/5 (0)
	2	UL		31	No fever–39.2		No	<64	0/5 (0)
	3	UL		32	No fever–39.4		No	<64	0/10 (0)
	4	UL		33	No fever–39.1		No	<64	0/11 (0)
	1	UL + IN		34	No fever–39.0		No	<64	0/5 (0)
	2	UL + IN		35	No fever–39.0		No	<64	0/8 (0)
	3	UL + IN		36	6–40.9		No	32,768	7/7 (100)
	4	UL + IN		37	No fever–38.9		No	<64	0/7 (0)
4	2	UL		38	No fever–38.8		No	<64	0/10 (0)
	2	UL + IN		39	10–40.1		No	32,768	10/10 (100)

*AI, acquisition infestation (see Figure and Tables 1–4); dpi, days postinfestation; IN, infected nymphs; ND, not done; UL, uninfected larvae. †See Figure and Tables 1–4. ‡Blood was collected at 21 days dpi and tested by IFA with *R. rickettsii* antigens. §This guinea pig died during the febrile period; its spleen was shown by real-time PCR to contain rickettsial DNA.

Nymphs from acquisition infestation 2, which fed as larvae on immune guinea pigs, were used to infest 12 guinea pigs (nos. 18–29). In every case in which the nymphs derived from engorged larvae that had fed alone in feeding chamber UL, no rickettsial transmission occurred (nos. 18–23). When nymphs derived from engorged larvae that had fed with *R. rickettsii* IN within the same chamber (UL + IN), absence of rickettsial transmission was demonstrated in 4 guinea pigs (nos. 24, 25, 28, 29), whereas rickettsial transmission was demonstrated by fever and seroconversion in 2 guinea pigs (nos. 26, 27). PCR on ticks demonstrated no rickettsial DNA in adult ticks that molted from engorged nymphs recovered from guinea pigs 18–25 and 28–29, which did not develop fever or serocont. On the other hand, rickettsial DNA was detected in most (80.0%–91.7%) of adult ticks that molted from engorged nymphs fed on guinea pigs 26 and 27, in which rickettsiosis developed.

We used nymphs from acquisition infestation 3, which fed as larvae on immune guinea pigs, to infest 8 guinea pigs (nos. 30–37). In every case where the nymphs derived from engorged larvae that had fed alone in feeding chamber UL, no rickettsial transmission occurred (guinea pigs 30–33). When nymphs derived from engorged larvae that had fed with *R. rickettsii* IN within the same chamber (UL + IN), absence of rickettsial transmission was demonstrated in 3 guinea pigs (nos. 34, 35, 37), whereas rickettsial transmission was demonstrated by fever and seroconversion in only guinea pig 36. PCR on ticks demonstrated no rickettsial DNA in adult ticks that molted from engorged nymphs recovered from guinea pigs 30–35 and 37. Conversely, we detected rickettsial DNA in 100% of adult ticks that molted from engorged nymphs fed on guinea pig 36, in which rickettsiosis developed.

Nymphs from acquisition infestation 4, which fed as larvae on a single immune guinea pig, were used to infest 2 guinea pigs (nos. 38, 39). When nymphs derived from engorged larvae that had fed alone in feeding chamber UL, no rickettsial transmission occurred. When nymphs derived from engorged larvae that had fed with *R. rickettsii* IN within the same chamber (UL + IN), rickettsial transmission was demonstrated by fever and seroconversion in guinea pig 39. PCR on ticks demonstrated no rickettsial DNA in adult ticks that molted from engorged nymphs recovered from guinea pig 38. Contrastingly, rickettsial DNA was detected in 100% of adult ticks that molted from engorged nymphs fed on guinea pig 39, in which rickettsiosis developed.

In summary, in all cases of rickettsial transmission by nymphs derived from engorged larvae that had fed on immune guinea pigs, the acquisition feeding was from the feeding chamber UL + IN, in which UL had fed with IN (guinea pigs 26, 27, 36, 39) (Table 5). In contrast, in the other 7 guinea pigs (nos. 24, 25, 28, 29, 34, 35, 37), which were infested with nymphs from acquisition feeding in chamber UL + IN, no rickettsial transmission occurred. We observed no rickettsial transmission in the 11 guinea pigs (nos. 18–23, 30–33, 38) that were infested by nymphs derived from engorged larvae that had fed physically separated from IN in feeding chamber UL on immune guinea pigs, even though a few of these nymphs contained rickettsial DNA after molting (Table 3, guinea pigs 3 and 4; Table 4, guinea pig 2).

We tested random samples of 5 real-time PCR–positive nymphs and adults from acquisition/transmission infestations by conventional PCR targeting a 532-bp fragment of the rickettsial *ompA* gene (*21*). PCR products were DNA sequenced and showed to be 100% identical to an *ompA* partial sequence of *R. rickettsii* from GenBank (accession no. KU321853).

## Discussion

Since the classical experiments of Ricketts (*6*), guinea pigs have been adopted as the animal model for *R. rickettsii* infection in the laboratory. In susceptible guinea pigs, fever typically develops a few days after infestation by *R. rickettsii*–infected ticks (*7**,**15**,**22**,**23*). This febrile period coincides with rickettsemia, as demonstrated by blood passages and rickettsial titration in guinea pig blood (*6**,**7**,**22**–**24*). During acquisition infestation 1 on susceptible guinea pigs 1–6, fever developed in all animals 5–9 dpi with *R. rickettsii*–IN. Because these febrile guinea pigs served as hosts for *A. aureolatum*–UL, we assume that these larvae fed on rickettsemic hosts. This condition explains the 100% PCR positivity for rickettsial DNA on nymphs that molted from these larvae, regardless of the feeding chamber (UL or UL + IN). This PCR positivity was confirmed by the transmission infestation with the molted nymphs, which in all cases transmitted *R. rickettsii* to susceptible guinea pigs. These results demonstrate horizontal transmission of *R. rickettsii* by co-feeding ticks on hosts with systemic *R. rickettsii* infection, which has been well known since Ricketts (*6*).

Guinea pigs from acquisition infestation 1 were exposed again to *R. rickettsii* IN during acquisition infestations 2–4, when fever did not develop in any animals. Based on their anti–*R. rickettsii* IgG titers at the infestation day, coupled with data reported in several previous studies (i.e., a previously infected animal will not develop a second rickettsemia [*7**–**9**,**22**,**23*]), we assume these animals were immune to *R. rickettsii* and did not develop rickettsemia during acquisition infestations 2–4. This statement was corroborated by the fact that none of the nymphs derived from larvae that fed alone (feeding chamber UL) transmitted *R. rickettsii* to susceptible guinea pigs (Table 5). These results demonstrate that horizontal transmission of *R. rickettsii* by co-feeding ticks on hosts with no systemic infection did not occur when UL fed distantly from IN.

When UL fed with *R. rickettsii* IN within the same chamber on immune guinea pigs (acquisition infestations 2–4), in most cases, nymphs that emerged from the engorged larvae were not able to transmit *R. rickettsii* to susceptible guinea pigs. However, in a few cases (guinea pigs 26, 27, 36, 39), rickettsial transmission occurred (fever, seroconversion). These results indicate horizontal transmission of *R. rickettsii* by co-feeding ticks on hosts with nonsystemic infection. We tested only infected donor nymphs with acquisition larvae. Further studies should evaluate the reverse approach—infected donor larvae with acquisition nymphs.

Overall, the transmission infestations were concordant with the PCR results on unfed nymphs before infestation; in every successful transmission of *R. rickettsii* to guinea pigs, specimens of the nymphal batch contained rickettsial DNA, as did the adults that molted from these nymphs. However, in 4 cases, the nymphal batch contained rickettsial DNA, but the nymphs did not transmit *R. rickettsii*; guinea pigs 28, 29, 32, and 33 received nymphs from batches in which 12%–16.7% of nymphs (derived from feeding chamber UL or UL + IN) contained rickettsial DNA, but none of the guinea pigs became infected by *R. rickettsii* (Table 5). These results highlight the weakness of PCR results alone when adopted to evaluate nonsystemic transmission of *R. rickettsii* among co-feeding ticks. Two previous studies proposed nonsystemic transmission of *R. conorii* between *Rhipicephalus sanguineus* ticks upon feeding on immune hosts (*25**,**26*); however, these studies relied solely on DNA detection in ticks after molting, and PCR results of postmolting ticks were not confirmed by transmission infestations. Our study convincingly demonstrates nonsystemic transmission of rickettsia by exposing postmolting-acquisition ticks to feed on susceptible hosts.

We created an artificial condition in which 200 acquisition larvae were limited to feed on a small area of the host skin (5 cm diameter) together with 50 *R. rickettsii* IN. This condition is unlikely to occur under natural conditions, where much lower number of larvae and nymphs are usually found feeding simultaneously on a small area of the skin. One reasonable explanation for nonsystemic transmission under such conditions, even though it occurred in only a few cases, was that acquisition ticks shared the same feeding site with IN on the host skin; therefore, they could have exchanged salivary secretions containing *R. rickettsii* of nymphal origin. This assumption is corroborated by the fact that all cases of nonsystemic transmission were from UL + IN feeding chambers. Taking into account that *R. rickettsii* infection rates in tick populations under natural conditions are typically very low (0.05%–1%) (*7**,**9**,**27**–**30*) and the low likelihood of ticks sharing the same feeding site, the importance of our results for the ecology of *R. rickettsii* could be insignificant. For example, although the systemic transmission tends to generate a great number of additional infected ticks (those feeding on every part of the host body during the few weeks of rickettsemia), the nonsystemic transmission might generate only 1 additional tick specimen, the one that, by chance, had fed side-by-side with the infected tick. The role of nonsystemic transmission to the ecology of *R. rickettsii* and other spotted fever group rickettsiae needs to be quantified in further studies.
